# Account of Diseases in an Eastern District of London, from October 20, to November 20, 1803

**Published:** 1803-12-01

**Authors:** 


### *Account of Diseases in an Eastern District of London, from October 20, to November 20, 1803.*

| | |
|---|---|
| ACUTE DISEASES. | |
| Pneumonia - - - - | 3 |
| Typhus Mitior - - - | 5 |
| Cholera - - - - - - | 12 |
| Dysenteria - - - - - | 6 |
| Hæmorrhagia Intestinalis | 1 |
| Rheumatismus Acutus - | 5 |
| CHRONIC DISEASES. | |
| Tussis - - - - - - | 10 |
| Tussis cum Dyspnœa - | 12 |
| Phthisis Pulmonalis - - | 3 |
| Hydrothorax - - - - | 4 |
| Ascites - - - - - - | 2 |
| Anasarca - - - - - | 4 |
| Gastrodynia - - - - | 5 |
| Enterodynia - - - - | 10 |
| Diarrhœa - - - - - | 14 |
| Dysuria - - - - - - | 3 |
| Menorrhagia - - - - | 2 |
| Amenorrhœa - - - - | 5 |
| Cephalalgia - - - - | 7 |
| Vertigo - - - - - - | 3 |
| Epilepsia - - - - - | 2 |
| Hemiplegia - - - - | 1 |
| Herpes - - - - - - | 1 |
| Rheumatismus Chronicus | 18 |
| PUERPERAL DISEASES. | |
| Ephemera - - - - - | 6 |
| Menorrhagia Lochialis - | 4 |
| Mastodynia - - - - | 3 |

Rhagas

| | | | |
|---|---|---|---|
| Rhagas Papillæ | 4 | Ophthalmia | 4 |
| INFANTILE DISEASES. | | Dentitio | 3 |
| Aphthæ | 7 | Convulsio | 2 |

The state of diseases during the last few weeks, does not afford an opportunity for any particular observations; it is sufficient to say, that it has been very similar to that which was included in the last Report. Complaints of the stomach and bowels have been very frequent, and have, as in former cases, been much connected with other morbid affections of the system.

